# Association mapping of autumn-seeded rye (*Secale cereale* L.) reveals genetic linkages between genes controlling winter hardiness and plant development

**DOI:** 10.1038/s41598-022-09582-2

**Published:** 2022-04-06

**Authors:** Monica Båga, Hirbod Bahrani, Jamie Larsen, Bernd Hackauf, Robert J. Graf, Andre Laroche, Ravindra N. Chibbar

**Affiliations:** 1 Department of Plant Sciences, University of Saskatchewan, Saskatoon, SK S7N 5A8 Canada; 2 Harrow Research and Development Centre, Agriculture and Agri-Food Canada, Harrow, ON N0R 1G0 Canada; 3 Institute for Breeding Research on Agricultural Crops, Julius Kühn-Institut, 18190 Sanitz, Germany; 4 Lethbridge Research and Development Centre, Agriculture and Agri-Food Canada, Lethbridge, AB T1J 4B1 Canada

**Keywords:** Genetics, Molecular biology, Plant sciences, Environmental sciences

## Abstract

Winter field survival (WFS) in autumn-seeded winter cereals is a complex trait associated with low temperature tolerance (LTT), prostrate growth habit (PGH), and final leaf number (FLN). WFS and the three sub-traits were analyzed by a genome-wide association study of 96 rye (*Secale cereal* L.) genotypes of different origins and winter-hardiness levels. A total of 10,244 single nucleotide polymorphism (SNP) markers were identified by genotyping by sequencing and 259 marker-trait-associations (MTAs;* p* < 0.01) were revealed by association mapping. The ten most significant SNPs (*p* < 1.49e−04) associated with WFS corresponded to nine strong candidate genes: *Inducer of CBF Expression 1* (*ICE1*), *Cold-regulated 413-Plasma Membrane Protein 1* (*COR413-PM1*), *Ice Recrystallization Inhibition Protein 1* (*IRIP1*), *Jasmonate-resistant 1* (*JAR1*), *BIPP2C1*-like protein phosphatase, *Chloroplast Unusual Positioning Protein-1* (*CHUP1*), *FRIGIDA*-like 4 (*FRL4-like*) protein, *Chalcone Synthase 2* (*CHS2*), and *Phenylalanine Ammonia-lyase 8* (*PAL8*). Seven of the candidate genes were also significant for one or several of the sub-traits supporting the hypothesis that WFS, LTT, FLN, and PGH are genetically interlinked. The winter-hardy rye genotypes generally carried additional allele variants for the strong candidate genes, which suggested allele diversity was a major contributor to cold acclimation efficiency and consistent high WFS under varying field conditions.

## Introduction

Rye (*Secale cereale* L.) is a member of the *Triticeae* tribe within the *Pooideae* subfamily, which belongs to the *Poaceae* grass family. Similar to the *Triticeae* crops barley (*Hordeum vulgare* L.) and bread wheat (*Triticum aestivum* L.), rye is mainly cultivated for its grain, but can also be grown for its biomass used as forage or production of bioenergy. The main rye producing areas are located in North America, China, Russia, and Europe, where the grain is used for production of bread, alcoholic beverages, and feed^1^.

The *Triticeae* genomes are likely derived from a common progenitor genome which explains the high sequence synteny between the the seven chromosomes of the diploid rye R genome (2n = 2x = 14), diploid H genome of barley and the three genomes (A, B, D) of hexaploid wheat^2^. Since the wheat and rye lineages diverged, several translocations have occurred within the rye genome as revealed by transcript mapping and genome sequence assemblies^2–4^. The haplotype diversity in rye is high^5^ as it is an open-pollinated species, whereas current wheat and barley cultivars have lower genetic diversity due to self-pollination and extensive breeding during the last century. Although some genetic erosion has occurred in improved rye germplasm, the landraces and wild rye lines display a relatively high allelic variation that is available for mining^6^.

Rye can serve as a good model for winter hardiness studies in *Triticeae* species, as many autumn-seeded rye varieties developed for northern latitudes have the highest winter-hardiness among cereal crops^7^. Most of the temperate grasses are inherently chilling tolerant, but acquire frost tolerance following weeks of acclimation to low but non-freezing temperatures^8^. For autumn-seeded cereals, the cold acclimation process occurs prior to winter and is characterized by two major physiological processes: (a) accumulation of increased freezing tolerance and (b) fulfilment of vernalization requirement causing shift from vegetative to reproductive growth at the shoot apical meristem (SAM). Upon vernalization saturation, further development of floral organs is paused until new growth from the crown tissue is stimulated by inductive photoperiod and temperature in the spring^9^.

Cold acclimation initiates when plants integrate and respond to environmental signals received from gradual reductions in photoperiod, daily temperature, light intensity, and red/far-red ratio in incoming light^10,11^. An early start and long acclimation period result in higher winter hardiness for winter cereals, and thus, the timing of floral transition impacts the total amount of accumulated freezing tolerance^12^. Cold acclimated cereals further increase their freezing tolerance when exposed to slightly below 0 °C temperatures, which stimulate a second hardening process^13^. Transcriptional analyses show different sets of genes are induced during the above-zero and sub-zero cold hardening processes^14^.

Winter-hardy plants undergo a multitude of developmental adjustments during cold acclimation to build up frost resistance^8^. The morphological changes observed in cereals generally include a switch to prostrate growth habit (PGH), compact growth, strengthening of cell walls, changes in membrane structures, and increased number of leaf initials formed at SAM^12,15–18^. The biochemical changes involve production of various cryoprotectants, antioxidants, and antifreeze proteins to aid protection against future frost damage^19,20^. An enhanced photosynthetic performance is observed for the most winter-hardy cereals and results in higher biomass production and reduced susceptibility to photoinhibition as compared to tender genotypes^21^. Through the modification of photosynthesis, winter-hardy cereals can effectively increase their carbon pools in crown tissues to be retrieved during deacclimation incidents throughout winter and regrowth in the spring. Cold acclimation also causes epigenetic changes at the DNA and histone levels with effects on gene activities^22,23^. Overall, cold responses are coordinated by an extensive cross-talk between cold-induced Ca^2+^, reactive oxygen species, phytohormone, and light signaling pathways^10,24–26^, of which retrograde/anterograde stress signaling between plastids and nucleus are suggested to play a central role^15,27^.

The *Vernalization 1* (*VRN1*) and *Frost resistance-2* (*FR-2*) loci on homoeologous group 5 chromosomes are the major determinants for freezing tolerance in *Triticeae* species^28^. Allelic differences at *VRN1*, *Fr-A2* and their interactions relate to cold acclimation efficiency and duration^29^. In addition, *VRN1 and FR-2* interactions with genes controlling plant development is required for optimal cold acclimation^12,30,31^. Large clusters of *AP2/ERF* transcription factor genes denoted *C-repeat Binding Factors* (*CBFs*) are present at *FR loci*, where variations in *CBF* expression levels, copy numbers, and haplotypes affect freezing tolerance and WFS levels in hexaploid wheat^32,33^, barley^34^ and rye^35,36^. Cold-induced CBFs bind to C-repeat/dehydration-responsive elements present in promoters of many cold-regulated (*COR*) genes and thereby induce their expression during cold stress^37^. Besides the *CBF-COR* regulon, additional pathways respond to cold stress^38^ as demonstrated by triple *CBF* mutants in *Arabidopsis* showing reduced frost tolerance but retained cold-response for several *COR* genes^39^.

In a previous study, we analyzed WFS in a rye population of 96 genotypes of different origins and winter hardiness levels and showed WFS was almost entirely determined by the cold acclimation process^40^. In addition, the developmental traits FLN and PGH were highly correlated with both WFS and LTT (r = 0.59–0.80; *p* < 0.001). Estimations of heritability for WFS, FLN and PGH revealed moderate to high values (*h*^2^ = 0.45 to 0.81), which allowed us in this study to perform a genome-wide association study (GWAS) with the aim to find genes influencing WFS and associated traits. GWAS is useful for identification of candidate genes for traits showing variation in a population of unrelated genotypes^41^. The genetic mapping technology has greatly benefitted from recently developed next generation sequencing and genotyping by sequencing (GBS) technologies, which allow identification of large numbers of single nucleotide polymorphism (SNP) markers^42^. Rye is well suited for association mapping as it’s out-crossing nature generates a low linkage disequilibrium (LD)^5^. In addition, a near full-length annotated genome (6.74 Gb of estimated 7.9 Gb) sequence is available for genome mapping^4^. The association study of rye genotype panel revealed several candidate genes were shared between WFS and one or more of the three associated traits; thus, suggesting the traits were genetically interconnected. The characteristics of nine strong candidate genes affecting WFS are discussed.

## Materials and methods

### Plant materials

Rye (*Secale cereale* L.) accessions (96 genotypes) used for association mapping are listed in Table S1 and were phenotyped in a separate study^40^. The genotypes were maintained through generations by crossing five plants per pollination bag. All experimental research and field studies on plants (cultivated or wild) including collection of plant material complied with the institutional and funding agencies, national and international guidelines and legislation. The permission to collect seeds and plant material was granted under the material transfer agreements with international gene banks and Universities.

### Field trials to determine WFS

Five years (2014–2019) of field trials were conducted at the University of Saskatchewan Experimental Farm, Saskatoon, Saskatchewan, Canada (52° 10′ N, 106° 30′ W, and 457 m altitude) as previously reported^40^. Briefly, the genotypes were seeded in early September with each genotype randomly placed in three 3.6 m rows, 100 seeds/row, and 30 cm row spacing. For each genotype and trial, the germination frequency was determined 6 weeks upon seeding and survival frequency was determined the following spring. The genotypes survival frequencies from the trials were used to calculated the Best Linear Unbiased Estimates (BLUEs)^43^ for WFS for each genotype using the statistical analysis Multi Environment Trial Analysis with R (META-R) software version 6.04 (CIMMYT Research Data & Software Repository Network, Mexico). Based on the BLUE scores, WFS for the population was divided into five groups: very high (64.5–92.5%; 19 genotypes), high (56.7–64.3%; 20 genotypes), moderate (46.2–56.6%; 19 genotypes), low (30.5–43.7%; 19 genotypes) and very low (0–25.2%; 19 genotypes) WFS (Table S1).

### Freeze tests to determine LTT

Freeze tests of cold acclimated plants were performed by a step wise decrease in temperature using an EPZ-4H test chamber (ESPEC North America Inc., Hudsonville, MI, USA) as described^40^. The freezing temperature at which 50% of the plants regrew two weeks upon exposure to freezing was noted as the LT_50_ temperature. Negative LT_50_ values represented the LTT score.

### Assessment of plant developmental traits

The two developmental traits PGH and FLN were analyzed in four separate trials with five plants per genotype in each trial as previously described^40^. PGH was rated upon completion of cold-acclimation in growth chamber. The visual PGH rating was based on three types of growth habits: (1) erect, (2) intermediate, and (3) prostrate. To determine FLN, plant leaves were labeled numerically as they developed from the primary stem. The flag leaf number was recorded as FLN. BLUE scores for the two developmental traits were calculated to determine the overall FLN and PGH scores for each genotype.

### DNA extraction and GBS analysis

Leaf tissue (~ 130 mg) was collected from five two-week-old plants of each genotype and ground to a fine powder in liquid nitrogen using a mortar and pestle. DNA was extracted from the homogenized leaf material using a standard hexadecetyltrimethylammonium bromide-based method. The concentration of isolated DNA was determined using a Quant-iT™ PicoGreen^®^ dsDNA Assay Kit (Invitrogen, Molecular Probes, Eugene, OR, USA). Uniform presence of high molecular weight DNA in the samples was confirmed by agarose (0.8%) gel electrophoresis. Normalized DNA preparations at 10 ng µL^−1^ (20 µL in total) were subjected to library preparations and GBS analysis, a service provided by Plateforme d’analyses Génomiques of the Institut de Biologie Intégrative et des Systèmes (IBIS), Université Laval, Québec, Canada. Reduced-representation libraries were prepared from *PstI* and *MspI* double-digested DNA as described^44^ using an Ion CHEF System, Hi-Q reagents, and P1 V3 chips (ThermoFisher Scientific, Life Technologies Inc., ON, Canada) for the library and chip preparations^45^. Sequencing analysis was done on an Ion Proton Sequencer (ThermoFisher Scientific, Life Technologies Inc., Waltham, MA, USA).

### SNP calling

Raw FASTQ files obtained from GBS analyses of the 96 rye genotypes were processed by Trimmomatic version 0.36^46^ with a four-base window setting and a minimum average quality score of 16. Barcodes, adaptors, and reads less than 36 bp were eliminated from the sequences before alignment to scaffold sequences of rye inbred line Lo7–version 2^3^ using Bowtie 2.0 version 2.2.6 software^47^. Duplicates were removed using the rmdp function^48^ and SNP variants including insertions/deletions (InDels) were detected by Sequence Alignment/Map tools version 0.1.19^48^. The markers were filtered using the Variant Call Format tool version 0.1.14^49^, a minor allele frequency > 5%, and a minimum reading depth of 6.0. A total of 10,244 SNP markers were identified and numbered according to matching scaffold of rye inbred line Lo7–version 2^3^. The selected markers contained no missing data. BLASTn analyses against the 2021 rye Lo7 v1 genome assembly^4^ were done using default settings and e-value threshold set to ≤ 1e−70. SNP marker distances and squared correlations of allele frequencies (r^2^) for 9547 mapped markers for the population were determined by the TASSEL version 5.2.20 software^50^. The generated data was displayed as linkage disequilibrium (LD) decay plots using the RStudio package version 3.5.1 software^51^. LD value at r^2^ = 0.20 was estimated from each plot’s trend line.

### Analyses of population structure

The genetic relationship among the 96 rye genotypes was analyzed using a model-based clustering method implemented by the STRUCTURE version 2.3.4 software^52^. The analysis was made using genetic information for all 10,244 SNP markers. Based on an admixed model and allele frequencies correlated, the number of subpopulations (K) ranging from 1 to 9 were tested with burn-in time set to 50,000 and Markov Chain Monte Carlo replication number to 100,000. A total of 20 runs were performed for each data set to compute the log likelihood for each simulated K value. The STRUCTURE HARVESTER version 0.6.94 software^53^ was used to establish the optimal number of sub-populations according to the ΔK method^54^.

To visualize the relative genetic distances between the rye genotypes, the SNP data was subjected to a principal component analysis (PCA) conducted using RStudio package version 3.5.1 software^55^. The evolutionary relationships among the rye genotypes were calculated by the centered JBS method provided by the TASSEL version 5.2.20 software ^50,56^, and the result was displayed as a neighbor-joining dendrogram tree.

### Marker-trait association (MTA) analysis

Phenotypic data for WFS, LTT, FLN, and PGH of the rye population was determined by replicated trials in a previous study^40^. The population structure obtained from STRUCTURE version 2.3.4, kinship data, genotype, and phenotype data for the rye population were used to determine *p* and R2 (explained variation) for MTAs by the mixed linear model available in TASSEL version 5.2.20 software^50^. The minor allele frequency was set to > 5%. Significant SNP variants were initially tested based on a false discovery rate (FDR)-adjusted *p* value of 0.05 following a step-wise procedure^57^, and lowest adjusted *p* values (threshold *p* < 1.49e−04) was identified after a Bonferroni correction^58^. To illustrate the relationships between the allele variations and trait values for the 96 genotypes, phenotype and genotype data for the population was subjected to a principal component analysis (PCA) using RStudio package version 3.5.1 software^55^. The results of the analyses were displayed as bi-plots.

### Prediction of candidate genes

The marker sequences carrying SNP of interest underwent BLASTN searches (default settings) against the *Secale cereale* Lo7 version 1 pseudomolecules hosted by Graingenes (https://wheat.pw.usda.gov/blast/) and the IPK Galaxy Blast Suite (https://galaxy-web.ipk-gatersleben.de) databases to identify matches to the latest assembly of rye Lo7 genome and its annotated genes^4^. BLAST searches were also made against the National Center for Biotechnology Information database (https://blast.ncbi.nlm.nih.gov). Gene orthologues in hexaploid wheat were identified by BLAST searches against the EnsemblPlants (http://plants.ensembl.org) wheat RefSeqv2.0 assembly constructed by the International Wheat Genome Sequencing Consortium^59^. The DNASTAR software package version 15.3.0 (DNASTAR. Madison, WI, USA) was used to analyze gene sequences. A physical map of rye was drawn using the MapChart software version 2.3^60^. Transmembrane, signal peptide and phosphorylation site predictions in proteins were done using TMHMM, SignalP 5.0, and NetPhos 3.1 programs available at https://services.healthtech.dtu.dk/.

### Consent for publication

The manuscript was reviewed, edited by all authors and approved for submission.

## Results

### Structure analysis of GBS data generated for rye population

The GBS analysis generated a total of 357.1 million reads with an average read length of 108 nucleotides. Alignment of the processed sequences to scaffold sequences of rye inbred line Lo7 genome version 2^3^ produced 252,158 SNP markers, of which 10,244 SNP markers with no missing data remained upon data filtering. BLASTn analyses of the marker sequences to the 2021 rye genome assembly^4^ assigned a total of 9,547 markers (93.2%) to chromosome positions. The markers were relatively evenly distributed on the genome, with an average of 1364 markers per chromosome (Table S2). Estimations of LD yielded values between 3428 and 5153 bp for the individual chromosomes and an average of 4266 bp for the whole genome (Table S2; Fig. S1). With relatively low LD values, the mapping data was considered suitable for association mapping.

**Figure 1:**
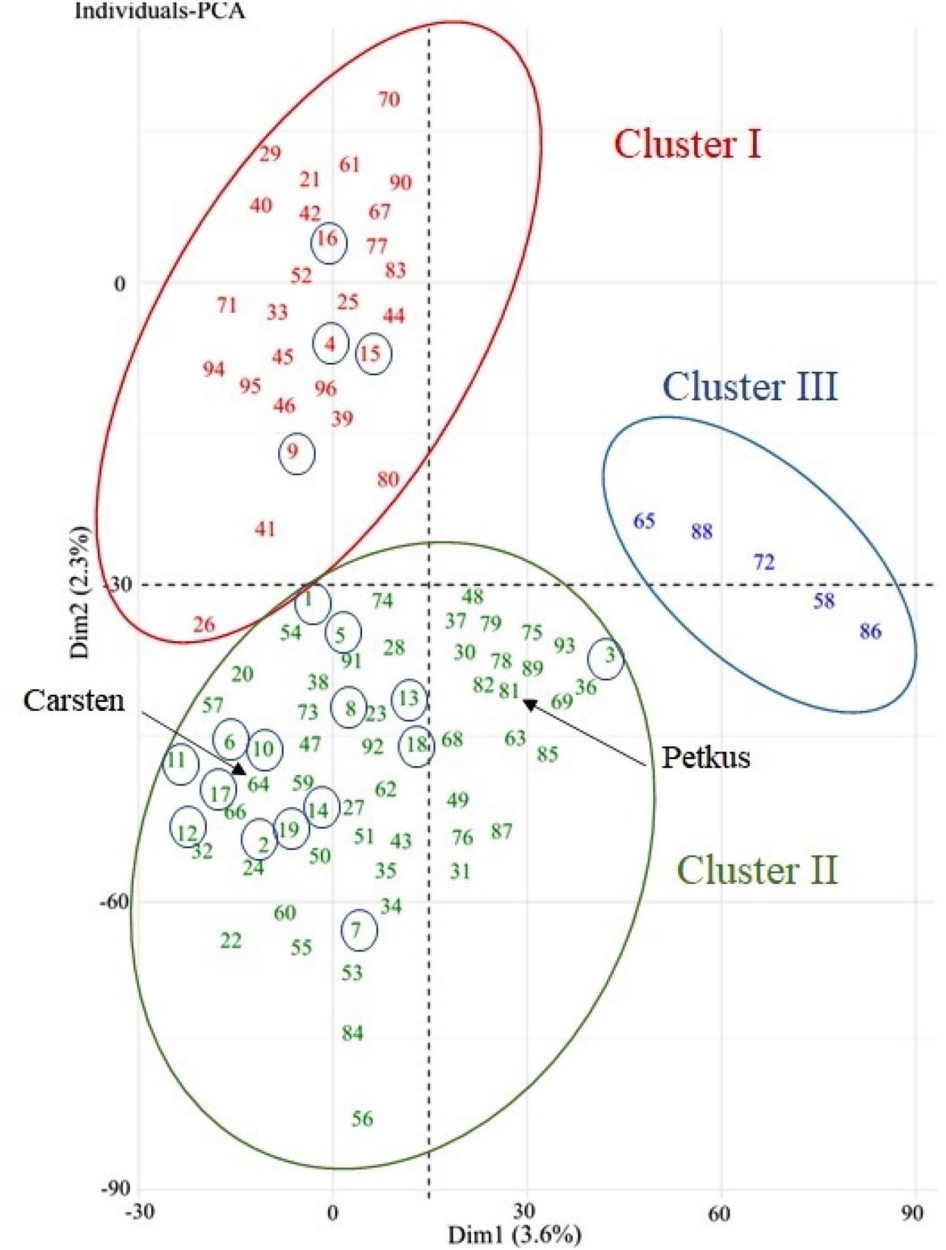
Principal component analysis (PCA) score plot. Plot generated from analysis of 10,244 SNP markers identified in rye population of 96 accessions. The x axis represents the eigenvalue for principal component 1 (PC1) and the y axis for PC2. Genotypes with highest WFS are encircled and arrows refer to less cold hardy Petkus and Carsten, respectively. Data for the individual genotypes is listed in Table S1.

The SNP data was analyzed by PCA, which showed the first and second coordinates explained 3.6% and 2.3% of the total genetic variation, respectively (Fig. 1). Three clusters were distinguished from the PCA plot, where the largest cluster (II) with 63 genotypes contained several genotypes with the highest WFS scores, but also relatively cold-sensitive genotypes such as Petkus and Carsten (Fig. 1; Table S1). Like cluster II, cluster I with 28 genotypes included several very winter-hardy genotypes intermingled with tender genotypes. Cluster III consisted mainly of perennial types with relatively low WFS scores. The existence of three subpopulations was also supported by a STRUCTURE HARVESTER analysis, which deduced the maximum value for Delta K to three (Fig. S2). A neighbor-joining phylogenetic tree constructed from the SNP data repositioned the Canadian genotypes Puma, Cougar and Gazelle in PCA cluster I to branch II of the phylogenetic tree (Fig. S3; Table S1). The tree also indicated division of branch II into two sub-branches, where Carsten was placed in branch IIa and Petkus in branch IIb. A separation of Carsten and Petkus into different gene pools can be explained by the use of these genotypes in different breeding programs^6,61^.

### Identification of SNP markers associated with developmental and cold tolerance traits

Association mapping using SNP and trait data for rye population led to identification of 189 SNP marker variations that were significantly (*p* < 0.01) associated with one or several of the WFS, LTT, FLN, and PGH trait values (Table S3). In total, 259 MTAs were identified, where 98 markers associated with WFS, 26 with LTT, 75 with FLN, and 60 with PGH (Table 1). LTT shared most markers with WFS (16 out of 26; 61.5%), followed by FLN (26 out of 75; 34.7%), and PGH (18 out of 60; 30.0%), thus indicating the four traits may be genetically linked with each other.

**Table 1:** Total number and shared SNP markers between traits.

Trait	Total number of SNPs associated with trait	Number of markers shared		
		LTT	FLN	PGH
WFS	98	16	26	18
LTT	26		9	9
FLN	75			13
PGH	60			

The SNP markers were divided into two groups based on their significance level (Table S3). Group 1 contained the ten most significant markers, which were distinguished after a Bonferroni correction of mapping data generating a new and more stringent significant *p* value (*p* < 1.49e−04; FDR < 0.05) (Table 2). A total of 22 MTAs was identified for the group 1 markers, which matched nine candidate genes. The second group included 179 markers (*p* < 0.001), of which 81 matched genes (Table S3). Of the 90 genes associated with MTAs, 32 amino acid substitutions were predicted from the SNP variations. All but five of the 189 SNP markers could be placed on de novo assembled Lo7 physical map of rye^4^ (Fig. 4). Below is a presentation of the nine strongest candidate genes listed in Table 2.

**Table 2:** Most significant MTAs identified in the study.

Marker ID^1/^	SNP^2/^	SNP position (bp)^3/^	*P *value^4/^	R2^5/^	MTA	Corresponding gene; protein	Amino acid change
Xuos526258	T/G	Chr7:112,789,055	1.10E−05	20.7	Xuos526258_WFS	*SECCE7Rv1G0469720.1*;Benzothiadiazole-induced protein phosphatase 2C1 (BIPP2C1)	V/G
			1.50 E−05	18.5	Xuos526258_FLN		
			3.84 E−05	16.4	Xuos526258_PGH		
			4.17 E−05	17.9	Xuos526258_LTT		
Xuos530120	G/C	Chr7:23,476,102	5.66 E−05	14.5	Xuos530120_WFS	*SECCE7Rv1G0458910.1*;Ice recrystallization inhibition protein 1 (IRIP1)	G/R
			6.62 E−05	16.3	Xuos530120_PGH		
			6.80 E−05	16.2	Xuos530120_LTT		
Xuos615052	T/A	Chr3:937,715,414	7.94 E−05	14.7	Xuos615052_WFS	*SECCE3Rv1G0209310.1*;Inducer of CBF expression 1 (ICE1)	L/H
			8.06 E−05	15.8	Xuos615052_FLN		
			8.12 E−05	14.7	Xuos615052_PGH		
			1.04 E−04	15.6	Xuos615052_LTT		
Xuos519455	T/A	Chr6:434,208,045	1.10 E−04	13.1	Xuos519455_WFS	*SECCE6Rv1G0399250.1*;Phenylalanine ammonia lyase 8 (PAL8)	F/I
Xuos613978	A/G	Chr5:727,383,264	1.11 E−04	15.5	Xuos613978_WFS	*SECCE5Rv1G0354870.1*, *SECCE5Rv1G0354880.1*;Cold-regulated plasma membrane 413 protein (COR413-PM)	T/A
		Chr5:727,387,758	1.18 E−04	15.3	Xuos613978_FLN		
Xuos75199	A/C	Chr1:716,318,047	1.20 E−04	15.3	Xuos75199_WFS	*SECCE1Rv1G0061510.1*;Jasmonat E−resistant 1 (JAR1)	N/T
			1.33 E−04	14.6	Xuos75199_FLN		
Xuos76228a	C/G	Chr2:933,364,125	1.39 E−04	14.5	Xuos76228a_WFS	*SECCE2Rv1G0140890.1*;Chalcone synthase 2 (CHS2)	Q/E
Xuos76228b	C/T	Chr2:933,364,201	1.44 E−04	13.4	Xuos76228b_WFS		A/V
Xuos2264	T/G	Chr1:109,131,359	1.45 E−04	12.5	Xuos2264_WFS	*SECCE1Rv1G0013960.1*;Chloroplast unusual positioning protein 1 (CHUP1)	None (G/G)
			1.45 E−04	14.4	Xuos2264_PGH		
			1.46 E−04	14.8	Xuos2264_FLN		
Xuos372616	C/A	Chr5:755,939,404	1.47 E−04	14.8	Xuos372616_WFS	FRIGIDA-like 4 (FRL4-like)	None (A/A)

^1/^Number refers to matching rye Lo7_v2_scaffold number^3^; ^2/^Reference/alternative allele; ^3/^Position on rye Lo7 pseudomolecules version 1 assembly^4^; ^4/^Calculated upon Bonferroni correction. ^5/^Explained variation.

### BIPP2C1-like protein phosphatase

**Figure 2:**
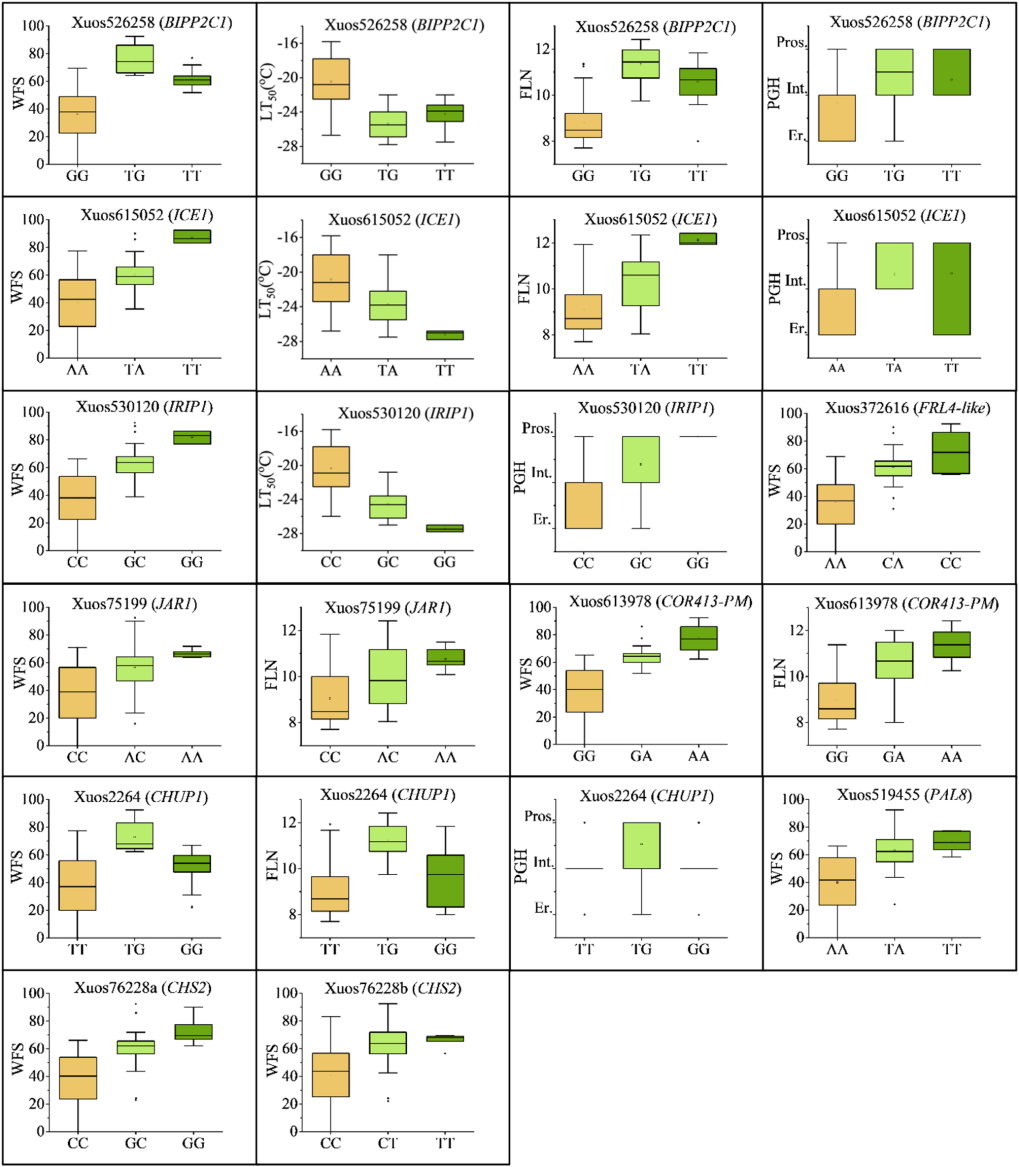
Box-whisker plots showing the allele effects for the most significant (*p* < 1.49 e−04) MTAs. The plots show median (horizontal bar), interquartile ranges (boxes), ranges (whiskers), and outliers (dots) for marker allele frequencies among the 96 rye genotypes.

The most significant marker identified in the study (Xuos526258) was associated with all four traits, WFS, LTT_,_ FLN, and PGH (Table 2) and matched *SECCE7Rv1G0469720.*1, a PPM-type protein phosphatase 2C gene (*PP2C*) located on chromosome 7R (112,789 Mb; Fig. 4). The predicted rye phosphatase showed highest sequence identity (84.1%) to benzothiadiazole-induced protein phosphatase 2C1 (*BIPP2C1*) from *Triticum dicoccoides* (XP_037426067). BIPP2C1 proteases are members of the Mg^2+^/Mn^2+^-dependent Ser/Thr phosphatase family subclade K in *Arabidopsis*, rice, and wheat^62–64^. No function has been assigned for wheat BIPP2C1^64^, whereas clade K member OsBIPP2C1/PP45 from rice is cold-induced^63^, protects against abiotic and biotic stresses^65^, and has a negative role in abscisic acid signaling^66^. The T/G SNP variation for rye *BIPP2C1* caused a V_185_ to G_185_ change within a glycine-rich region located outside of the catalytic domain for the phosphatase (data not shown). All 38 rye genotypes with low or very low WFS encoded only the G_185_ variant for the phosphatase (Table S4), and this allele variant was also associated with low FLN values, low LTT (high LT_50_ values), and erect growth habit (Fig. 2). Genotypes encoding both (V_185_/G_185_) or only V_185_ variant for BIPP2C1 had overall the highest WFS, LTT and FLN values (Fig. 2; Table S4).

### Ice recrystallization inhibition protein 1 (IRIP1)

The high-significance marker Xuos530120 associated with WFS, LTT, and PGH (Table 2) was found to match *SECCE7Rv1G0458910.1* located on chromosome 7R (23,476 Mb; Fig. 4). The targeted gene codes for an ice recrystallization inhibition protein 1 (IRIP1), which belongs to a group of proteins only produced by *Pooideae* species^67^. IRIPs carry a N-terminal leucine-rich repeat (LRR) domain, similar to those of phytosulfokine receptor tyrosine kinases, and an ice-binding domain at the C-terminal end^68^. During exposure to freezing, IRIPs present in the apoplastic space bind to small ice crystals and thereby inhibit further ice recrystallization and frost damage of the plasma membrane^67,69^. The proteins may also prevent bacteria from serving as ice-nucleators in the extra-cellular space^70^. The Xuos530120 SNP encoded a G_36_/R_36_ variation within one of two preserved cysteine loops preceding the LRR-like region, which may be proteolytically separated from the ice-binding domain in the apoplastic space as suggested by studies in *Brachypodium distachyon*^69^. Thus, the amino acid substitution for the rye IRIP was not expected to influence protein binding to ice crystals, but could affect the function of the N-terminal region (Fig. S4). Almost all genotypes (36 out of 38) with low and very low WFS encoded only the R_36_ IRIP variant (Table S4), whereas the highest WFS, LTT, and FLN values were favored by the G_36_ IRIP variant (Fig. 2). Many additional IRIP variants may be produced from the 7R locus as the reference genome carries at least 13 *IRIP-*like coding regions (> 80% nucleotide sequence identity) arranged in tandem with *SECCE7Rv1G0458910*.

### Cold-regulated 413 plasma membrane (COR413-PM)-like

One common SNP for WFS and FLN was carried by marker sequence Xuos613978 (Table 2), which showed high sequence identity to two *COR413* genes, *SECCE5Rv1G0354870.1* (89.1%) and *SECCE5Rv1G0354880.1* (89.2%), respectively. The two *COR* genes are arranged in tandem ~ 11 Mb distal of the vernalization locus (*ScVRN1*) on chromosome 5R (727,383 and 727,388 Mb; Fig. 4). COR413 genes are expressed during cold stress and their expression levels relate to accumulated freezing tolerance in cereals and *Arabidopsis* during cold acclimation^71,72^. The rye COR413 proteins encoded from 5R locus were from BLAST and SignalP 5.0 algorithm analyses predicted to be targeted to the plasma membrane (Fig. S4). Additional predictions by TMHMM algorithm supported the N-terminal end of COR413-PM encoded by *SECCE5Rv1G0354870.1* was positioned inside of the plasma membrane, whereas the N-terminal end of COR413-PM encoded from *SECCE5Rv1G0354880.1* was positioned outside of the plasma membrane (Fig. S4). The Xuos613978 SNP caused a T_30_ to A_30_ variation proximal of the first predicted transmembrane region on *COR413-PM*. The SNP variation may involve a possible phosphorylation site at T_30_ according to NetPhos 3.1 analysis. Genotypes with low or very low WFS encoded only the A_30_ variant for COR413-PM, which was also associated with low FLN (Fig. 2; Table S4). High WFS and FLN values were associated with the T_30_ modification for COR413-PM (Fig. 2; Table S4).

### Inducer of CBF expression 1 (ICE1)

Marker Xuos615052 associated with WFS, LTT, FLN and PGH (Table 2) coincided with an *Inducer of CBF Expression 1* (*ICE1*) gene (*SECCE3Rv1G0209310.1*) positioned on chromosome 3R (937,715 Mb; Fig. 4). Like the related ICE2, ICE1 is a MYC-like basic helix-loop-helix (bHLH) transcription factor, which binds to *MYC*-recognition sequences present in *CBF* promoter regions and thereby stimulate cold-induced *CBF* expression^73,74^. Induced CBFs then activate cold-dependent and abscisic acid-independent expression of *COR* genes conferring cold tolerance in plants^37^. *SECCE3Rv1G0209310.1* showed 97% sequence identity to orthologous *TaICE41* on chromosomes 3D in hexaploid wheat, which contributes to increased freezing tolerance when overexpressed in *Arabidopsis*^75^. The Xuos615052 SNP caused a L_283_/H_283_ variation at the end of the DNA-binding bHLH domain and start of a ZIP dimerization domain based on domain structures predicted for ICE proteins^76^ (Fig. S4). Similar to the rye H_283_ ICE1 variant, a basic residue (K) is present at corresponding site for ICE1 isoforms XI and X2 produced by *Aegilops tauschii* subsp. *tauschii* (XP_020168495 and XP_020168503), *AenICE2* of *Aegilops neglecta*, and *AeuICE2* of *Aegilops umbellulata*, respectively^77^. Among 38 rye genotypes with low or very low WFS, 33 encoded only the H_283_ ICE1 variant (Table S4), which was also associated with low LTT, low FLN, and erect growth habit (Fig. 2). Most (24 out of 39) rye genotypes with very high and high WFS encoded only L_283_ or both variants (L_283_/H_283_) for ICE1 (Fig. 2; Table S4).

### Jasmonate-resistant 1 (JAR1)

Two markers, Xuos75199 (Table 2) and Xuos75046 (Table S3), both associated with WFS and FLN, targeted the same gene, (*SECCE1Rv1G0061510.1*) on chromosome 1R (716,318 Mb; Fig. 4). The *SECCE1Rv1G0061510.1* transcript showed strong sequence identity (95%) to an *Aegilops tauschii* subsp. *tauschii*, *Jasmonate-resistant 1* (*JAR1*) mRNA isoform X3. JAR1 is a cytosolic auxin-inducible enzyme catalyzing reversible conversion of Jasmonic Acid (JA) to JA-Ile, which is the active growth regulator in JA signaling^78^. Both JA and methyl jasmonate (MeJA) can be converted to JA-Ile and participate in the activation of systemic defense; thus, JA is involved in several stress responses^79^. The more significant SNP for *JAR1* (Xuos75199) caused an N_11_/T_11_ variation, of which the T_11_ variant was common among genotypes with low or very low WFS (29 out of 38 genotypes; Table S4) and associated with low FLN values (Fig. 2). JAR1 encoded by genotypes with high and very high WFS generally carried alleles coding for both (N_11_/T_11_) variants (Fig. 2; Table S4).

### FRIGIDA-like 4 (FRL4-like)

Marker Xuos372616 associated with WFS corresponded to *SECCE5Rv1G0358510.1* located on chromosome 5R (755,939 Mb; Table 2; Fig. 4) and coding for a FRIGIDA-like protein based on BLASTp searches. High sequence identities were also noted to a C-terminal FRIGIDA-like domains carried by 5'-3' exoribonuclease 4 (XRN4) proteins encoded by *Brachypodium distachyon* (XP_024313537), *Oryza sativa* (XP_025879725), and *Triticum urartu* (EMS54431.1). Interestingly, *SECCE5Rv1G0358500.1* located 1,343 bp upstream and oriented in the same direction as *SECCE5Rv1G0358510.*1 codes for a XRN4-like protein without the FRIGIDA-like domain. As evidence is lacking for a rye transcript coding for both XRN4 and FRIGIDA-like domains, we assume the gene targeted by Xuos372616 only encoded the FRIGIDA-like protein. Amino acid alignment to FRIGIDA-like proteins characterized in *Arabidopsis*^80^ revealed the rye FRIGIDA-like protein had highest similarity (59–61%; data not shown) to subfamily IV represented by AtFRL4a and AtFRL4b. *AtFRL4b* is co-regulated with genes encoding chromatin remodeling factors and proteins involved in floral transition^81^ and maize *FRL4* also participates in a chromatin remodeling network involving MADS box factors controlling floral development^82^. However, no information regarding *FRL4* action among *Triticeae* species is available. For the rye FRL4-like protein, the Xuos372616 SNP did not alter the encoded amino acid (A_235_). Homozygous A/A alleles at SNP site dominated among genotypes with low or very low WFS (36 out of 38), whereas both (C/A) alleles were frequent among genotypes with very high and high WFS (21 out of 39). (Fig. 2; Table S4). *FRL4*-like gene seems a reasonable candidate gene for Xuos372616; however, it cannot be excluded that the upstream *SECCE5Rv1G0358500.1* encoding XRN4 or a protein with both XRN4 and FRIGIDA-like domains is the actual gene causing the trait variance.

### Chloroplast unusual positioning protein-1 (CHUP1)

Two of the markers identified in the study coincided with genes controlling chloroplast movement. Marker Xuos2264 associated with WFS, FLN, and PGH was of higher significance (Table 2) and corresponded to *SECCE1Rv1G0013960.1* gene positioned on chromosome 1R (109,131 Mb; Fig. 4). This gene codes for a Chloroplast Unusual Positioning Protein-1 (CHUP1), but the Xuos2264 SNP variation T/G did not alter the protein sequence at G_2_. Genotypes with low and very low WFS generally carried the T allele at SNP position, whereas both SNP variants were preferred by genotypes with high WFS (Table S4). The second chloroplast movement marker (Xuos370689) was only significant for WFS (Table S3) and matched *SECCE5Rv1G0331080.1* positioned on chromosome 5R (536.242 Mb; Fig. 4). The Xuos370689 marker targeted a *WEAK CHLOROPLAST MOVEMENT UNDER BLUE LIGHT 1* (*WEB1*) gene, for which SNP variation (A/G) was synonymous (R_310_/R_310_). CHUP1, which is localized in the chloroplast envelope, regulates polymerization and/or maintenance of chloroplast actin (cp-actin), which mediates blue light-regulated translocation of chloroplasts to specific sites on the plasma membrane depending on the intensity of incoming light^83^. WEB1 has a role in blue-light-induced reorganization of cp-actin filaments during the avoidance response and is associated with the speed chloroplast move^84^. The identification of highly significant SNPs targeting two different genes involved in chloroplast relocation suggest positioning of chloroplasts could be an important factor for WFS in rye.

### Phenylalanine Ammonia-lyase 8 (PAL8) and Chalcone Synthase 2 (CHS2)

Among the highly significant markers for WFS were markers for genes encoding enzymes in the phenylpropanoid pathway. A Phenylalanine Ammonia-lyase 8 (PAL8) gene (*SECCE6Rv1G0399250.1*) located on chromosome 6R (434.208 Mb; Fig. 4; Table 2) was targeted by marker Xuos519455 and a Chalcone Synthase 2 (CHS2) gene (*SECCE2Rv1G0140890.1*) by two markers (Xuos76228a and Xuos76228b) on chromosome 2R (933,364 Mb; Fig. 4; Table 2). PAL is the first enzyme in the general phenylpropanoid pathway and catalyzes channeling of *L*-Phe from the primary metabolism to synthesis of *trans*-cinnamic acid. This enzymatic step constitutes the starting point for numerous polyphenol compounds such as flavonols, stilbenes, lignin, and anthocyanins in plants^85^. At the Xuos519455 SNP site for PAL8, a F_291_/I_291_ variation was encoded. The alternative I_291_ variant was very common in genotypes with low and very low WFS (35 out of 38), whereas the reference F_291_ allele was primarily encoded by the most winter-hardy genotypes (Fig. 2; Table S4). CHS2 acts downstream of PAL and diverts compounds away from the general phenylpropanoid pathway and towards flavonoids by catalyzing condensation of three malonyl-CoA molecules with one 4-coumaroylCoA molecule to form naringenin chalcone. The Xuos76228a SNP caused a Q_79_/E_79_ variation and Xuos76228b SNP an A_104_/V_104_ variation for CHS2 predicted from *SECCE2Rv1G0140890.1*. The reference Q_79_ and A_104_ residues were favored by genotypes with low and very low WFS (34 out of 38 genotypes; Fig. 2; Table S4), whereas the alternative E_79_ and V_104_ residues were most common among genotypes with very high WFS (Fig. 2; Table S4).

### Variation for CBF genes showed low significance for WFS

In this study, three markers significant for WFS matched CBF genes at *FR-R2* on chromosome 5R, but at lower significance level (*p* < 0.01; Table S3) than those listed in Table 2. The genes targeted by markers Xuos369733, Xuos390086, and Xuos370362 were *CBFIIId-19* (*SECCE5Rv1G0340540.1*), *CBFIVa-2.2* (*SECCE5Rv1G0340460.1*), and *CBFIIIa-6.2* (*SECCE5Rv1G0340660.1*), respectively (5R:614.963–616.449 Mb; Fig. 4). SNP variations for *CBFIIId-19* were synonymous (D_401_/D_401_), whereas SNP variation for *CBFIVa-2.2* caused an H_113_/P_113_ change within encoded AP2 DNA-binding region and immediately prior to signature motif CMIII-1 (Fig. S4). SNP variation for *CBFIIIa-6.2* resulted in a L_224_ to H_224_ variation within CBFIIIa-6.2 C-terminal region (Fig. S4). A preference for the alternative alleles for *CBFIVa-2.2* (P_113_), and *CBFIIIa-6* (H_224_) was found for genotypes with low or very low WFS (data not shown). Besides *CBFs* at *FR-A2*, Xuos328096 marker associated with FLN matched *SECCE4Rv1G0278790.*1 encoding CBFII*-5* from chromosome 4R (810.923 Mb; Fig. 4). The SNP at *CBFII-*5 caused an R_37_ to P_37_ change within conserved signature sequence CMIII-3 (Fig. S4), which is essential for *Arabidopsis CBF1* transcriptional activity^86^. Whether any of the amino acid variations identified for CBFIVa-2.2, CBFIIIa-6, or CBFII-5 affected CBF function in rye remains to be demonstrated.

### Bi-plot analysis supports WFS and associated traits are controlled by several common factors

**Figure 3:**
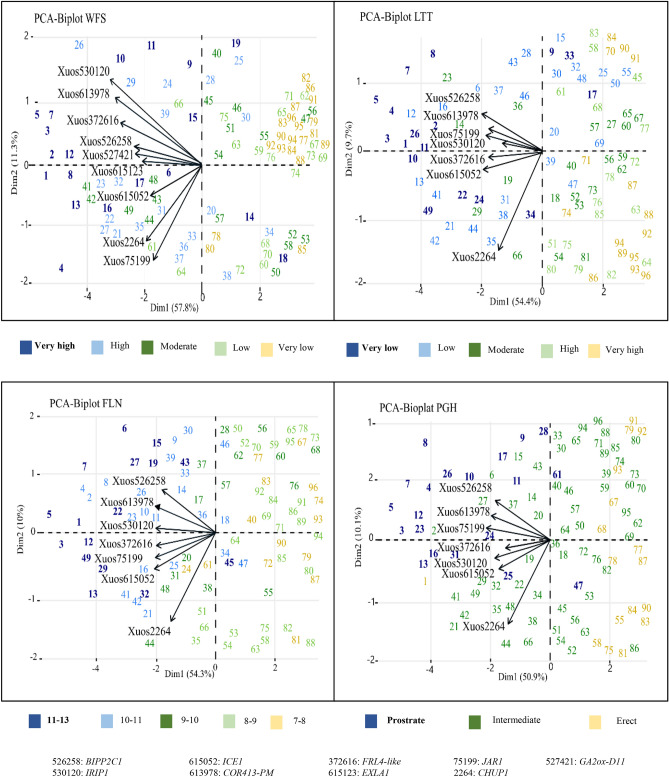
PCA bi-plot visualizing SNP effects on four traits analyzed for rye population of 96 genotypes.

**Figure 4:**
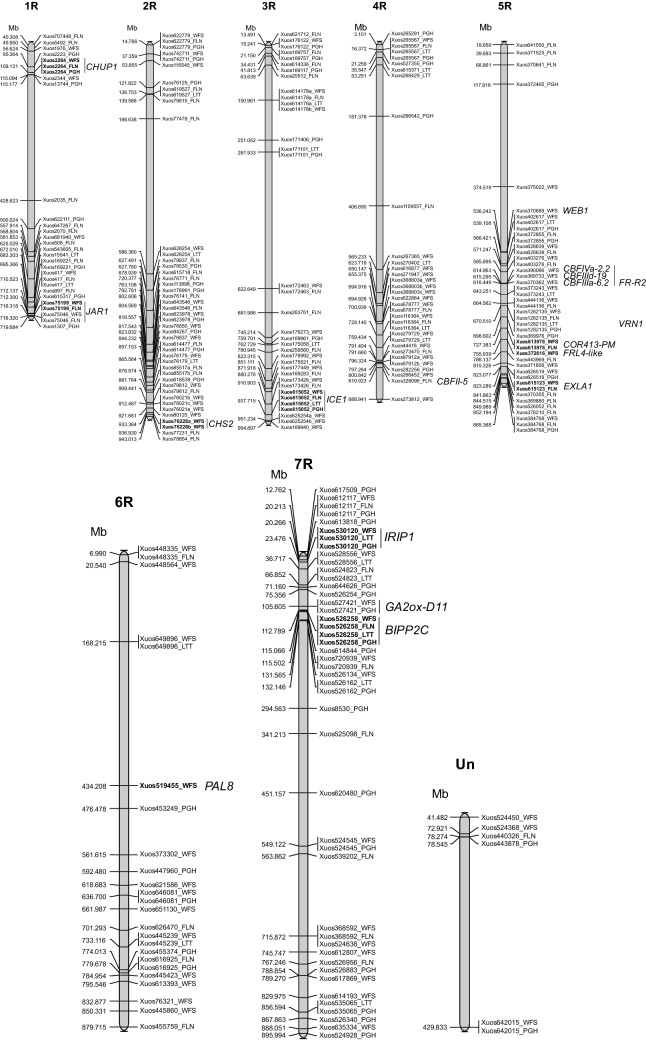
Location of significant MTAs identified on rye physical map. Locations refers to the Lo7 v1 pseudomolecules 2021^4^. Most significant markers (*p* < 1.49e−04; Table 2) are indicated in bold. Location of *FR-R2*, *VRN1*, and discussed candidate genes are shown.

The four traits analyzed in the study were analyzed by PCA, which revealed relatively high amount of trait variation explained by PC1 (50.9 to 57.8%) and PC2 (9.7 to 11.3%) in the four trait bi-plots (Fig. 3). In all bi-plots, genotypes with very high and high WFS were mostly found in the first and fourth quadrants, whereas genotypes with low WFS were located in the second and third quadrants. Seven vectors representing SNP markers for candidate genes *IRIP1*, *COR413-PM*, *FRL4*-like, *JAR1*, *ICE1*, *BIPP2C1*, and *CHUP1* were visualized for each of the LTT, FLN, and PGH bi-plots, which all showed similar patterns (Fig. 3). In these three bi-plots, the first six vectors correlated relatively well with each other and contributed mainly to PC1, whereas *CHUP1* vector showed a different direction and was mainly associated with PC2. The WFS bi-plot was also represented by *IRIP1*, *COR413-PM*, *FRL4*-like, *ICE1*, and *JAR1* vectors, but in contrast to the other bi-plots, the *JAR1* vector was oriented closer to the CHUP1 vector. WFS was also represented by two additional vectors (markers Xuos527421, Xuos615123; Table S3) corresponding to gibberellin 2-beta-dioxygenase D11 (GA2OX-D11; 7R:105.61 Mb) and expansin-like A1 (EXLA1; 5R:823.29 Mb) genes, respectively (Fig. 4). Both WFS and PGH were associated with *GA2OX-D11* (Xuos527421_WFS; Xuos527421_PGH; Table S3), which encodes an enzyme deactivating gibberellins and stimulating of compact growth^87^, which is generally displayed by winter-hardy cereals. Expansins have a role in cell wall loosening causing changes to cell wall elasticity and are generally associated with stress responses affecting plant growth; however, the exact function for *EXLA1* is not known^88^.

## Discussion

### Rye, a model for study of winter hardiness in cereals

The LD decay rate is the major factor limiting mapping resolution in GWAS and it is largely determined by the mating system (self or out-crossing), recombination rate, distribution of recombination hot spots on the genome, and population structure^89^. In this respect, rye with a low average intra-genic LD of about 4.3 kb (Table S2) or less^5^ is well suited for association mapping, which provides a considerable higher mapping resolution than bi-parental mapping^41^. In this study, the mapping precision was further improved by using a reduced-representation GBS library targeting low-copy hypo-methylated regions for SNP identification^44^. Successful accomplishment of this strategy was demonstrated by 90 out of 189 (47.6%) significant SNP markers mapped to annotated genes (Table S3).

The PCA and STRUCTURE analyses grouped the rye population of 96 genotypes into three main subpopulations (Figs. 1, S1, S2; Table S1). Many of the winter-hardy Canadian rye cultivars were grouped together with genotypes with relatively low winter-hardiness levels (Table S1). Overall, the clustering of rye genotypes did not relate well to their geographical origin or WFS values (Table S1), but a slight preference around the Carsten genepool was indicated for genotypes with very high WFS (Fig. 1; Table S1). Although breeding has reduced the genetic pool of rye^6^, it has still been possible to select/develop very cold hardy rye varieties with winter hardiness far exceeding that of wheat and barley cultivars, which have undergone loss of diversity for genes conferring high WFS. Thus, the key genetic factors required to improve winter hardiness in tender winter cereals are present in certain rye genotypes.

### Adjustment of photosynthesis during cold acclimation

Allele differences for *CHUP1* and *WEB1* encoding proteins involved in chloroplast movement^83,84,90^ were associated with WFS levels within the rye population (Table S3). *CHUP1* allele variation was also suggested by the PCA bi-plot analysis to affect PGH, FLN, and LTT trait values (Fig. 3), which are determined during the cold acclimation process. Chloroplast movement to the periclinal cell wall is suggested to enhance photosynthesis and biomass production under low light conditions, whereas movement to anticlinal cell wall was initially postulated to protect the photosynthetic apparatus from high light^91^. Later studies suggest the avoidance reaction fine-tunes light signals or regulates signaling pathways^90^. Chloroplasts have a very important role during cold acclimation in winter cereals, as they adjust the photosynthesis efficiency upon response to environmental temperature and light signals^11,92^. This allows certain cold-hardy winter cereals to increase their photosynthesis efficiency during cold acclimation when compared to less winter-hardy genotypes^21^. A study in Arabidopsis supports the photosynthetic acclimation depends on chloroplast relocation which coordinates photosynthesis with down-stream carbohydrate metabolism^93^. Retrograde and anterograde signaling during cold stress may also be affected by chloroplast repositioning as nuclei attach and co-migrate with chloroplasts to new destinations on the plasma membrane^94^. Although no amino acid difference was associated with SNP variations for *CHUP1* or *WEB1*, it is possible these genes are differentially regulated by light and/or temperature signals in rye genotypes with varying degree of WFS. Further analysis of rye CHUP1 and WEB1 genes and expression levels during cold acclimation are needed to confirm their role during cold acclimation in rye.

### Protection of membrane integrity during cold acclimation

Like the organelle membranes, the plasma membrane is prone to freezing injury and must be protected to withstand cell dehydration caused by apoplastic freezing and membrane fracturing by growing ice crystals. The protection mechanisms generally include membrane rigidification^95^, production of proteins with membrane stabilization properties^96^, and prevention of ice crystal formation and growth^97^. One of the cold-induced plasma membrane proteins is the multi-spanning COR413-PM, for which sequence variation was associated with WFS and FLN (Table 2). Overexpression of *PsCOR413PM2* isolated from *Phlox subulata* in *Arabidopsis* causes an increased Ca^2+^ flux in roots and higher expression of *CBF1, CBF3* and certain *COR* genes not induced by CBFs^98^. Thus, *COR413* acts early in the cold-induction pathway and both *COR413-PM1* and related chloroplast *COR413-IM* in *Arabidopsis* are included in a set of 56 plant core environmental stress genes triggering systemic stress responses; however, the systemic signal inducing this response is not known^99^. Although the precise COR413-PM function has not been determined, it has been suggested the membrane-bound protein maintains membrane fluidity at low temperature^100^, acts as a G protein-coupled receptor involved in signaling^72^, or stabilizes the membrane lipid structure^98^.

Like COR413-PM, IRIPs are important for membrane protection. In this study, WFS, LTT, and PGH for the rye population were strongly associated with allelic variation for one of many IRIP genes encoded from a 7R locus (Table 2). IRIPs contribution to frost-hardiness is supported by an increased *IRIP* expression during cold acclimation in wheat^101^ and improved freezing tolerance in transgenic *Arabidopsis* expressing *LpIRI-a*, *LpIRI-b*, *LpIRI2*, or *LpIRI3* from *Lolium perenne*^102^. A reduced freezing tolerance caused by down-regulation of *IRIPs* in *Brachypodium distachyon* further confirms ice-binding proteins contribute to development of frost tolerance in cereals^69^. Prevention of ice crystal growth in the apoplast is particularly critical during freeze/thaw cycles, which may occur during winter or early spring for winter cereals.

Unexpectedly, our study suggested IRIP sequence variation affected PGH (Table 2), which is caused by curvature of shoots emerging from the crown during cold acclimation. The phenotype is likely due to altered gravitropic responses affecting auxin distribution at the shoot base^103^. The shoot is one of first plant tissues that freezes, despite the lower part of the plant is warmer than the upper part^68^. Thus, very cold hardy cereals have evolved a more efficient frost protection for SAM than less cold-hardy genotypes to assure survival of the crown during winter and regrowth in the spring. The association between IRIP1 variation and PGH suggests the ice-binding proteins are produced at SAM; however, there is no information regarding expression levels of *IRIP* genes in rye crowns. In wheat, JA and ethylene activate *TaIRIP-1* expression in leaves, crowns, and roots during cold acclimation, but the two phytohormones do not activate *TaIRIP-2*, which is only expressed in leaves^67^. Thus, studies of tissue-specific expression of *IRIP* alleles are needed to reveal which IRIP isoform(s) encoded from 7R affects PGH and possibly SAM development during cold acclimation.

### Candidate genes belonging to the ICE-CBF-COR regulon

Like other *Triticeae* members, rye *Fr-R2* carries a cluster of *CBF* genes^104^, for which haplotype variation is suggested to underlie differences in freezing tolerance among rye accessions^4,36^. However, the significant associations between WFS and three different *CBF* genes (*CBFIIId-D19*, *CBFIVa-2.2*, and *CBFIIIa-6*) at *FR-R2* were not among the strongest MTAs (Table S3). Transcript analysis of rye *CBF* genes indicates existence of three *CBFIVa-2.*2 and two *CBFIIIa-6* genes^105^. This could possibly explain the low significance for individual *CBF* alleles as complementary functions may be exerted by additional copies of near-identical *CBF*; thus, SNP variations for multi-copy *CBF* genes contribute little to phenotype differences within the population. Lack of strong association between SNP variations for individual *CBF* genes at *Fr-A2* and frost resistance is also seen in a panel of 1739 European winter wheat genotypes studied by association mapping^106^. However, most mapping studies of bi-parental populations in wheat, barley, and rye show *Fr-2* has a major role for frost tolerance^35,107,108^. Due to the limited number of recombination events and narrow genetic variation within the populations studied, the bi-parental confidence intervals for QTL regions are large, and for the *FR* loci on group five chromosomes, defined by a composite effect from several *CBF* genes, their expression levels, interactions, and copy number effects.

Although variation for individual *CBF* genes appeared to have a minor role for WFS, several candidate genes belonging to the ICE-CBF-COR regulon were identified. One strong candidate gene was *ICE1* (Table 2), for which amino acid variation at the end of the DNA binding bHLH region and/or start of zipper region was associated with WFS, LTT, FLN and PGH (Fig. S4). In a previous study, allele variation within an intron for *ScICE2*, probably allelic with *ICE1* identified in this study, was associated with variation for winter hardiness and freezing tolerance among 201 European rye genotypes^36^. Thus, specific *ICE* alleles may be important for winter hardiness in rye. Since genotypes with low or very low WFS generally encoded ICE1 containing the rare H_283_ modification, these ICE1 proteins may display reduced affinity for the MYC recognition elements (CANNTG) present in promoters of *CBF* genes and several other cold-regulated genes^73,74^. Alternatively, the H_283_ ICE1 variation, which is also located at the start of the ZIP domain, could affect dimer formation^76^. Through heterodimers formed with various bHLH factors, both ICE1 and ICE2 have a role in the establishment and differentiation of stomata in dicot plants^109^ and stomata lineage formation in monocot plants^110^. Through similar heterodimer formations, additional developmental roles for rye ICE1 may exist at SAM as *ICE1* allele variation in this study was associated with both FLN and PGH besides WFS and LTT (Table 2).

### Study suggests jasmonic acid influences cold acclimation and floral transition

Allele variations for *JAR1* was associated with WFS and FLN in our study (Table S3). In support of *JAR1* causing variation for WFS in rye are previous studies demonstrating JA has a role in the development of cold stress resistance in *Arabidopsis*^26,111^. These studies show cold-induced JA-Ile production leads to activation of the *ICE*-*CBF*-*COR* regulon, which could explain the association between *JAR1* and WFS observed in our study (Table 2). The association between *JAR1* and FLN (Table 2) further suggested JA-Ile influences timing of vegetative to reproductive transition at SAM during cold acclimation. Such a role for *JAR1* is supported by studies in maize and rice associating JAR1 activity with delayed juvenile-to-adult transition at SAM in both species^112,113^. During vernalization in wheat, the MeJA levels are shown to increase, but they decline post-vernalization when plants return to normal temperature^114^. An increased expression of *VRN1* and *VRN3* and delayed flowering is demonstrated by exogenous application of MeJA to spring wheat, which suggests JA regulates vernalization requirement and modulates flowering time in wheat^114^. Thus, it is possible the association between *JAR1*, WFS and FLN observed in our study involves *JAR1*-mediated regulation of *VRN1* with effects on timing of floral transition.

Another JA-induced effect during cold acclimation in *Arabidopsis* is biosynthesis of cold-protective secondary metabolites, such as polyamines, glutathione, and anthocyanins^26,115^. The production of glycosylated cyanins in particular is associated with enhanced winter hardiness in rye population analyzed in this study^116^. Thus, allele variations for *JAR1* and two of the genes encoding early enzymes in the phenylpropanoid pathway, PAL8, CHS2 (Table 2), may have a role in increasing anthocyanin levels during cold acclimation in several of the most winter-hardy genotypes. JA can also affect cold acclimation independent of the CBF pathway whereby growth is regulated^117^. Thus, *JAR1* can have many different roles during and after cold acclimation, which may be reflected in the different positions of the *JAR1* vector in the WFS bi-plot versus the LTT, FLN, PGH bi-plots (Fig. 3).

### Expansion of stress related alleles in winter-hardy rye

Rye genotypes with high and very high WFS showed an overall higher frequency of allele variants for candidate genes than genotypes with low or very low cold hardiness (Fig. 3; Table S4). This tendency was particularly obvious for *IRIP1*, *COR413-PM*, *FRL4-like*, *BIPP2C1*, *CHUP1*, *CHS2* and *PAL8*. The trend suggested the most winter-hardy genotypes have evolved higher fitness to winter conditions through acquisition of additional alleles for some of the important cold-responsive genes. A wide-spread duplications of rye genes as compared to other cereals is supported by a recent near full-length (98.47%) genome assembly of Chinese rye line Weining with a low heterozygosity rate of 0.26%^118^. Similar to the expansion of *CBF* and *IRIP* gene families that occurred when Pooideae lineages adapted to extreme temperatures during the Eocene–Oligocene transition^68,119^, the extra alleles for some of the cold-regulated candidate genes identified in this study may have evolved during periods of cold stress in the history of rye. Gene expansion is not unique to cereals, but has occurred throughout the evolutionary history of plants and is predominantly observed for stress-responsive genes^120^. Whether the additional gene copy number for cold-responsive genes identified by our study provides that extra winter-hardiness observed for some of the Canadian rye cultivars when compared to other rye genotypes needs to be explored further.

## Concluding remarks

The phenotypic relationships reported earlier for the rye population studied here showed that sub-traits like LTT, FLN, and PGH with high heritability estimates (*h*^2^ = 0.45 to 0.81), can be used to dissect a complex trait like WFS^40^. With the abundance of SNP markers provided by GBS combined with a draft genome sequence for rye, the association mapping conducted on the rye population identified in total 90 annotated candidate genes (*p* < 0.01). The accuracy of the mapping is strengthened by finding seven of the nine strongest candidate genes have roles in plant adaptation during cold acclimation and contribute to enhanced winter hardiness in cereals and other plants. For two strong candidate genes, *BIPP2C* and *FRL4-like*, there are no obvious associations to WFS in *Triticeae* species. As similar genes in other monocot species are implicated in floral transition, studies of the rye *BIPP2C* and *FRL4-like* is likely to advance our knowledge of the cold tolerance process in winter cereals. Thus, future studies are planned with a diverse panel of rye genotypes with varying LTT and/or WFS to validate all the candidate genes identified in this study, many of which seem promising but were not discussed here. The long-term objective is to verify gene variants that enhance winter hardiness in rye and use this information to increase WFS in temperate winter cereals of interest for the Northern hemisphere. Identification of genetic factors contributing to WFS in winter cereals is of particular importance considering the immediate challenges of global warming increasing the demand for crops with higher resilience to variable weather patterns.

## Supplementary Information

Supplementary Information 1.

Supplementary Information 2.

Supplementary Information 3.

Supplementary Information 4.

Supplementary Information 5.

Supplementary Information 6.

Supplementary Information 7.

Supplementary Information 8.

Supplementary Information 9.

## Data Availability

All data used in the study is included in the manuscript and in the supplementary data files.
