# Supplementary material for: Association mapping of autumn-seeded rye (Secale cereale L.) reveals genetic linkages between genes controlling winter hardiness and plant development
Source: Sci Rep. 2022 Apr 6;12:5793. doi: 10.1038/s41598-022-09582-2 (PMC8986816; doi:10.1038/s41598-022-09582-2)
Supplement: Supplementary file 1 — Supplementary Information 1. [file 41598_2022_9582_MOESM1_ESM.docx]

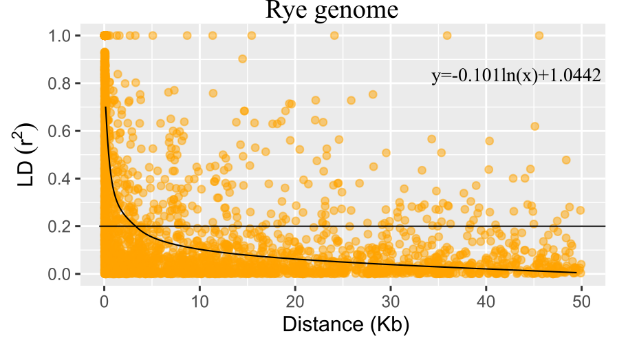

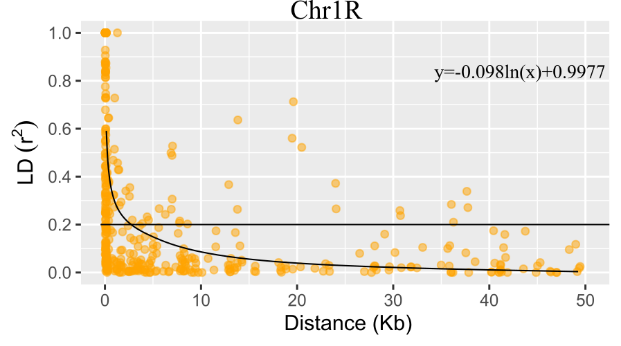

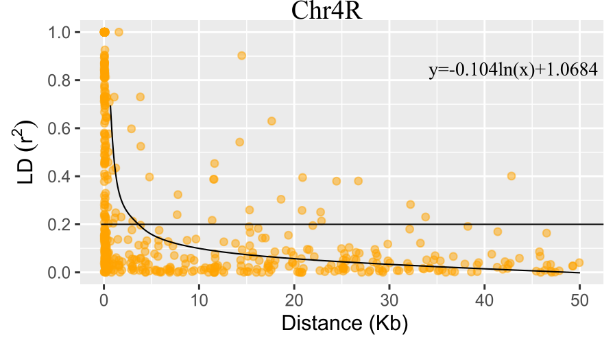

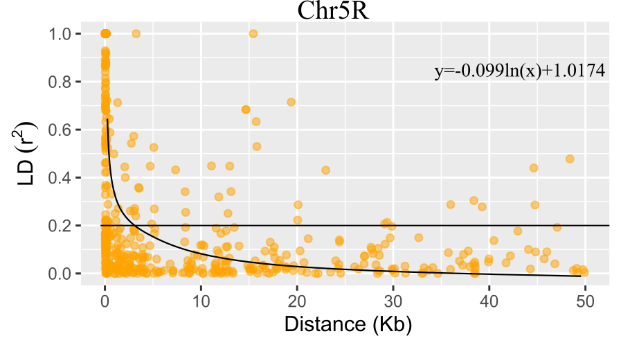

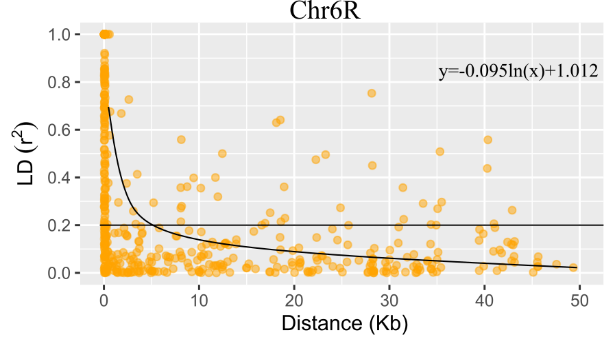

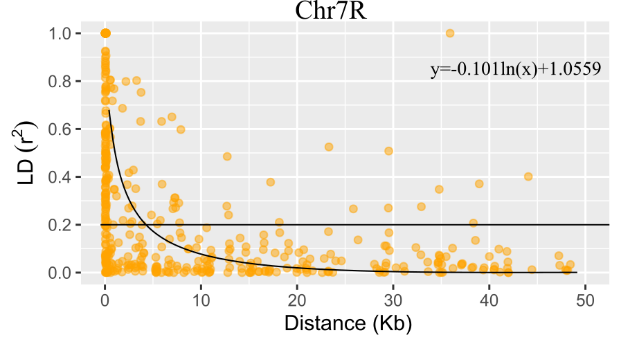

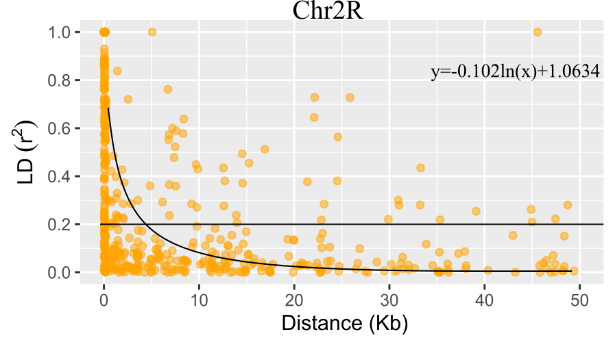

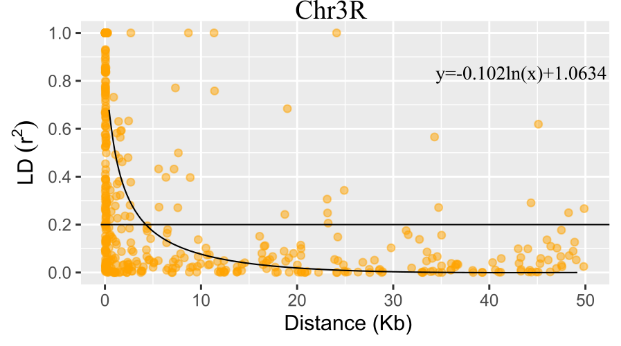


**Fig. S1.** Linkage disequilibrium (LD) decay plots across rye chromosomes and whole genome. The plots were based on SNP markers (9,547 in total) for 96 rye accessions and show the squared correlation coefficient between SNP markers (r^2^) on the y-axis and physical distance (kb) between SNP markers on the x-axis. LD was estimated at r^2^ = 0.20.
