# Supplementary material for: Association mapping of autumn-seeded rye (Secale cereale L.) reveals genetic linkages between genes controlling winter hardiness and plant development
Source: Sci Rep. 2022 Apr 6;12:5793. doi: 10.1038/s41598-022-09582-2 (PMC8986816; doi:10.1038/s41598-022-09582-2)
Supplement: Supplementary file 2 — Supplementary Information 2. [file 41598_2022_9582_MOESM2_ESM.docx]

Cluster 2


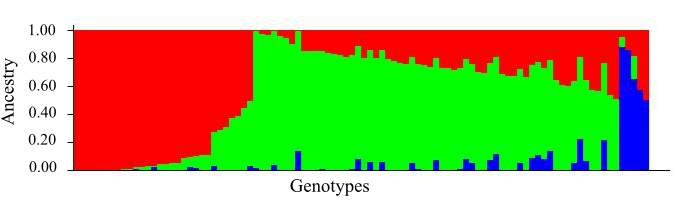


Cluster 1

Cluster 3

**Fig. S2.** STRUCTURE plot for rye population. Plot was based on analysis of 10,244 SNP markers identified for 96 rye accessions. Each genotype is represented by a vertical line, for which colored segments indicate estimated fractions of three (ΔK=3) ancestral populations predicted for the population.
