# Supplementary figures and images for: Association mapping of autumn-seeded rye (Secale cereale L.) reveals genetic linkages between genes controlling winter hardiness and plant development
Source: Sci Rep. 2022 Apr 6;12:5793. doi: 10.1038/s41598-022-09582-2 (PMC8986816; doi:10.1038/s41598-022-09582-2)

**IIb**

Cluster I


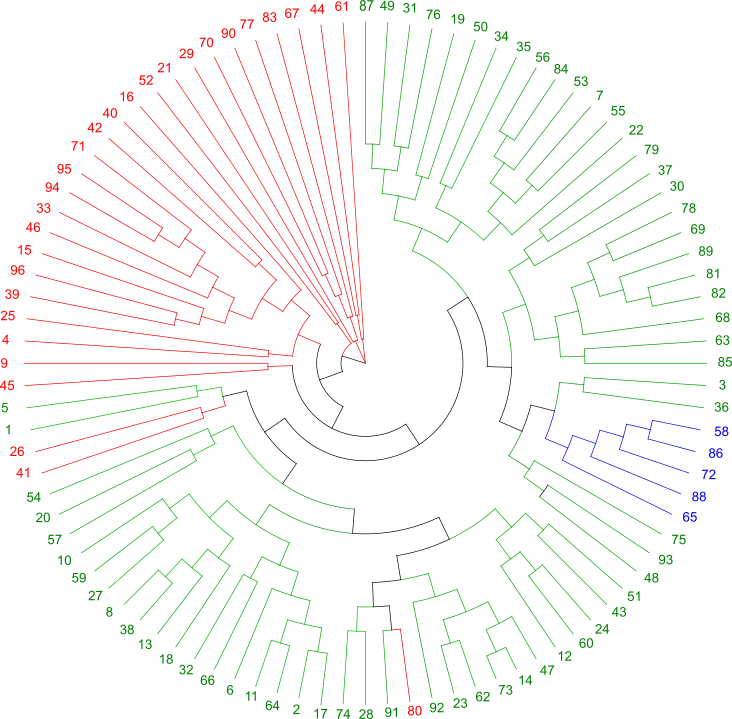


Cluster III

Cluster II

**I**

**IIa**

Supplement: Supplementary file 3 — Supplementary Information 3. [file 41598_2022_9582_MOESM3_ESM.docx]
