# Supplementary material for: Association mapping of autumn-seeded rye (Secale cereale L.) reveals genetic linkages between genes controlling winter hardiness and plant development
Source: Sci Rep. 2022 Apr 6;12:5793. doi: 10.1038/s41598-022-09582-2 (PMC8986816; doi:10.1038/s41598-022-09582-2)
Supplement: Supplementary file 4 — Supplementary Information 4. [file 41598_2022_9582_MOESM4_ESM.docx]

**Fig. S4.** Illustration indicating location of variants amino acids in proteins encoded by six candidate genes for WFS.
