# Supplementary material for: Association mapping of autumn-seeded rye (Secale cereale L.) reveals genetic linkages between genes controlling winter hardiness and plant development
Source: Sci Rep. 2022 Apr 6;12:5793. doi: 10.1038/s41598-022-09582-2 (PMC8986816; doi:10.1038/s41598-022-09582-2)
Supplement: Supplementary file 5 — Supplementary Information 5. [file 41598_2022_9582_MOESM5_ESM.docx]

| **Table S1.** Rye accessions classified based on winter field survival and genotype clustering. | | | | | | |
| --- | --- | --- | --- | --- | --- | --- |
| **Genotype** | **Origin** | **Growth habit** | **WFS   BLUE score^1^** | **PCA cluster^2^** | **NJtree branch^3^** |  |
|  |  |  | **Very high:** |  |  |  |
| 1-Leth Coulee Rye | Canada | Winter | 92.5 | II | IIa |  |
| 2-Gauthier | Canada | Winter | 90.1 | II | IIa |  |
| 3-AC Remington | Canada | Winter | 86.2 | II | IIb |  |
| 4-AC Rifle | Canada | Winter | 85.9 | I | I |  |
| 5-Musketeer | Canada | Winter | 83.0 | II | IIa |  |
| 6-SM 38R | Canada | Winter | 77.5 | II | IIa |  |
| 7-Prima | Canada | Winter | 77.0 | II | IIb |  |
| 8-Saratovskaja 4 | Russia | Winter | 71.8 | II | IIa |  |
| 9-SM 4R | Canada | Winter | 71.0 | I | I |  |
| 10-Pearl | Denmark | Winter | 69.5 | II | IIa |  |
| 11-Kustro | Canada | Winter | 68.8 | II | IIa |  |
| 12-Kharkivska 95 | Ukraine | Winter | 67.9 | II | IIa |  |
| 13-Kharkivska 98 | Ukraine | Winter | 66.9 | II | IIa |  |
| 14-Esprit | Germany | Winter | 66.3 | II | IIa |  |
| 15-Ponsi | Sweden | Winter | 66.0 | I | I |  |
| 16-Hazlet | Canada | Winter | 65.5 | I | I |  |
| 17-Antelope | Canada | Winter | 65.3 | II | IIa |  |
| 18-Emerald | USA | Winter | 65.2 | II | IIa |  |
| 19-Anna | Finland | Winter | 64.5 | II | IIb |  |
|  |  |  | **High:** |  |  |  |
| 20-R003-4 | Canada | Winter | 64.3 | II | IIa |  |
| 21-Voima | Finland | Winter | 64.2 | I | I |  |
| 22-Dakota | Canada | Winter | 64.1 | II | IIb |  |
| 23-Sc-73 | Canada | Winter | 64.0 | II | IIa |  |
| 24-Animo | Netherlands | Winter | 63.6 | II | IIa |  |
| 25-Caribou | Canada | Winter | 63.6 | I | I |  |
| 26-Puma | Canada | Winter | 62.4 | I | IIa |  |
| 27-Othello | Sweden | Winter | 62.2 | II | IIa |  |
| 28-Rymin | USA | Winter | 61.9 | II | IIa |  |
| 29-Adams | USA | Winter | 61.5 | I | I |  |
| 30-Sangaste | Estonia | Winter | 60.3 | II | IIb |  |
| 31-Visa | Finland | Winter | 59.9 | II | IIb |  |
| 32-Vitallo | Germany | Winter | 59.6 | II | IIa |  |
| 33-Halo | Germany | Winter | 59.5 | I | I |  |
| 34-Balbo | Italy | Facultative | 59.4 | II | IIb |  |
| 35-Frontier | Canada | Winter | 58.6 | II | IIb |  |
| 36-Enzi | Finland | Winter | 58.4 | II | IIb |  |
| 37-Explorer | USA | Facultative | 58.4 | II | IIb |  |
| 38-Motto | Poland | Winter | 58.0 | II | IIa |  |
| 39-Dankowskie Selekcyjne | Poland | Winter | 56.7 | I | I |  |
|  |  |  | **Moderate:** |  |  |  |
| 40-Galma | Belgium | Winter | 56.6 | I | I |  |
| 41-Cougar | Canada | Winter | 56.1 | I | IIa |  |
| 42-Dominant | Netherlands | Winter | 55.8 | I | I |  |
| 43-Dankowskie Nowe | Poland | Winter | 54.9 | II | IIa |  |
| 44-Danko | Canada | Winter | 54.2 | I | I |  |
| 45-ACE-1 | Canada | Perennial | 54.0 | I | I |  |
| 46-Dankowskie Srebrne | Poland | Winter | 53.9 | I | I |  |
| 47-Carolkurz | Germany | Winter | 53.2 | II | IIa |  |
| 48-Horton | Canada | Winter | 53.1 | II | IIb |  |
| 49-Kodiak | Canada | Winter | 51.8 | II | IIb |  |
| 50-GC-100 | Russia | Winter | 51.6 | II | IIb |  |
| **Table S1**. cont. | | | | | |  |
| 51-Amilo | Poland | Winter | 49.2 | II | IIa |  |
| 52-Sellino | Germany | Winter | 48.5 | I | I |  |
| 53-R538 | UK | Perennial | 48.1 | II | IIb |  |
| 54-Protector | Germany | Winter | 47.8 | II | IIa |  |
| 55-Toivo | Finland | Winter | 47.5 | II | IIb |  |
| 56-Culpan | Russia | Winter | 47.0 | II | IIb |  |
| 57-Hardy white spring rye | Austria | Winter | 46.9 | II | IIa |  |
| 58-Maton | USA | Facultative | 46.2 | III | IIb |  |
|  |  |  | **Low:** |  |  |  |
| 59-Stoir | Ukraine | Winter | 43.7 | II | IIa |  |
| 60-Vaschod | Belarus | Winter | 43.7 | II | IIa |  |
| 61-R550 | Czech Rep. | Perennial | 43.6 | I | I |  |
| 62-Reimann Philipp | Germany | Perennial | 42.4 | II | IIa |  |
| 63-Oklon | USA | Facultative | 40.9 | II | IIb |  |
| 64-Carsten | Germany | Winter | 39.5 | II | IIa |  |
| 65-R903 | unknown | Perennial | 38.9 | III | IIb |  |
| 66-Harach | Canada | Spring | 38.8 | II | IIa |  |
| 67-Danae | Germany | Winter | 37.1 | I | I |  |
| 68-Clse 35 | USA | Winter | 36.8 | II | IIb |  |
| 69-Gator | USA | Facultative | 36.0 | II | IIb |  |
| 70-Elbon | USA | Facultative | 35.9 | I | I |  |
| 71-L-286-R | Germany | Winter | 35.7 | I | I |  |
| 72-R904 | Unknown | Perennial | 35.4 | III | IIb |  |
| 73-Syn 20-L | Germany | Winter | 35.3 | II | IIa |  |
| 74-SR4A-S5 | Canada | Spring | 33.2 | II | IIa |  |
| 75-Dakold | USA | Winter | 31.1 | II | IIb |  |
| 76-Wheeler | USA | Winter | 31.0 | II | IIb |  |
| 77-M.Karlic CT2 | Russia | Winter | 30.5 | I | I |  |
|  |  |  | **Very low:** |  |  |  |
| 78-Wintergrazer 70 | USA | Facultative | 25.2 | II | IIb |  |
| 79-Petkus Kurzstroh | Germany | Winter | 24.1 | II | IIb |  |
| 80-Gazelle | Canada | Spring | 23.6 | I | IIa |  |
| 81-Petkus | Germany | Winter | 22.9 | II | IIb |  |
| 82-Prolfic Spring | Canada | Spring | 22.1 | II | IIb |  |
| 83-Wren Abruzzi | USA | Facultative | 20.0 | I | I |  |
| 84-Extra Early Rye1 | Mexico | Spring | 19.7 | II | IIb |  |
| 85-Somro | Germany | Winter | 16.0 | II | IIb |  |
| 86-R1210 | South Africa | Perennial | 15.7 | III | IIb |  |
| 87-Baltia | Russia | Winter | 15.6 | II | IIb |  |
| 88-R797 | Poland | Perennial | 13.2 | III | IIb |  |
| 89-Fl-Synt | USA | Spring | 12.9 | II | IIb |  |
| 90-Ottawa Select | Canada | Winter | 12.9 | I | I |  |
| 91-Gulzow Kunz CT1 | Germany | Winter | 12.4 | II | IIa |  |
| 92-Rogo | Germany | Spring | 12.4 | II | IIa |  |
| 93-Florida 401 | USA | Spring | 7.1 | II | IIb |  |
| 94-L-145-N | Germany | Winter | 0.0 | I | I |  |
| 95-L-145-P | Germany | Winter | 0.0 | I | I |  |
| 96-L-18-R | Germany | Winter | 0.0 | I | I |  |

^1/^ Winter survival class based on BLUE scores^40^. ^2/^ see Fig. 1. ^3/^ see Fig. S2.
