# Supplementary material for: Association mapping of autumn-seeded rye (Secale cereale L.) reveals genetic linkages between genes controlling winter hardiness and plant development
Source: Sci Rep. 2022 Apr 6;12:5793. doi: 10.1038/s41598-022-09582-2 (PMC8986816; doi:10.1038/s41598-022-09582-2)
Supplement: Supplementary file 6 — Supplementary Information 6. [file 41598_2022_9582_MOESM6_ESM.docx]

| Table S2. Genomic distribution and LD of SNPs physically mapped to rye genome. | | | |
| --- | --- | --- | --- |
| Chromosome | **Number of SNPs*** | **SNP distribution on pseudomolecules (bp)**** | **Linkage disequilibrium (LD) (bp)** |
| ScLo7-21-Chr1R | 1,183 (11.5 %) | 819,198-726,669,444 | 3,428 |
| ScLo7-21-Chr2R | 1,427 (13.9 %) | 1,444,859-945,635,129 | 4,744 |
| ScLo7-21-Chr3R | 1,283 (12.5 %) | 924,802-964,932,827 | 4,744 |
| ScLo7-21-Chr4R | 1,495 (14.6 %) | 833,833-905,721,637 | 4,230 |
| ScLo7-21-Chr5R | 1,475 (14.4 %) | 94,483-875,796,416 | 3,853 |
| ScLo7-21-Chr6R | 1,423 (13.9 %) | 161,138-885,150,550 | 5,153 |
| ScLo7-21-Chr7R | 1,261 (12.3 %) | 25,042-899,480,522 | 4,790 |
| All Chr | 9,547 (93.2 %) |  | 4,266 |
| ScLo7-21-ChrUn | 416 (4.1 %) | 321,978-515,529,233 |  |
| Not mapped | 281 (2.7 %) |  |  |
| * A total of 10,244 SNP markers were analyzed. ** 2021 Lo7 v2 rye genome assembly^4^. | | | |
