# Supplementary material for: Association mapping of autumn-seeded rye (Secale cereale L.) reveals genetic linkages between genes controlling winter hardiness and plant development
Source: Sci Rep. 2022 Apr 6;12:5793. doi: 10.1038/s41598-022-09582-2 (PMC8986816; doi:10.1038/s41598-022-09582-2)
Supplement: Supplementary file 9 — Supplementary Information 9. [file 41598_2022_9582_MOESM9_ESM.docx]

**Association mapping of autumn-seeded rye (*Secale cereale* L.) reveals genetic linkages between genes controlling winter hardiness and plant development.**

**Monica Båga^1^, Hirbod Bahrani^1^, Jamie Larsen^2^, Bernd Hackauf^3^, Robert J Graf^4^, Andre Laroche^4^ and Ravindra N Chibbar^1*^**

**Supplementary information.**

**Fig. S1.** Linkage disequilibrium (LD) decay plots across rye chromosomes and whole genome. The plots were based on SNP markers (9,547 in total) for 96 rye accessions and show the squared correlation coefficient between SNP markers (r^2^) on the y-axis and physical distance (kb) between SNP markers on the x-axis. LD was estimated at r^2^=0.20.

**Fig. S2.** STRUCTURE plot for rye population. Plot was based on analysis of 10,244 SNP markers identified for 96 rye accessions. Each genotype is represented by a vertical line, for which colored segments indicate estimated fractions of three (ΔK=3) ancestral populations predicted for the population.

**Fig. S3.** Neighbor-joining phylogenetic tree for rye population. The tree is based on standard genetic distance with 10,000 individual bootstraps and numbers refer to genotypes listed in Table S1. Clusters identified by PCA analysis (Fig. 1) are indicated by colors.

**Fig. S4.** Illustration indicating location of variants amino acids in proteins encoded by six candidate genes for WFS.

**Table S1.** Rye accessions classified based on winter field survival and genotype clustering.

**Table S2.** Genomic distribution and LD of SNPs physically mapped to rye genome.

**Table S3.** All significant MTA identified by GWAS of rye population.

**Table S4.** Allele distribution for 10 SNP markers strongly associated with WFS in rye population of 96 genotypes. Genotypes are grouped according to their WFS level.
